# Maternal trauma and fear history predict *BDNF* methylation and gene expression in newborns

**DOI:** 10.7717/peerj.8858

**Published:** 2020-05-22

**Authors:** Stefanie R. Pilkay, Terri Combs-Orme, Frances Tylavsky, Nicole Bush, Alicia K. Smith

**Affiliations:** 1 School of Social Work, Syracuse University, Syracuse, NY, United States of America; 2 College of Social Work, University of Tennessee—Knoxville, Knoxville, TN, United States of America; 3 Department of Preventive Medicine, University of Tennessee—Memphis, Memphis, TN, United States of America; 4 Department of Psychiatry, University of California, San Francisco, CA, United States of America; 5 Department of Pediatrics, University of California, San Francisco, CA, United States of America; 6 Department of Gynecology and Obstetrics, Emory University, Atlanta, GA, United States of America

**Keywords:** Trauma, Epigenetic, BDNF, Intergenerational, Fear

## Abstract

Trauma and related fear exert significant influence on mental and physical health throughout the lifespan and are associated with intergenerational patterns of development, health, and behavior. DNA methylation and gene expression are involved in our developmental adaptations to our experiences and can be influenced by social interventions. Patterns of DNA methylation and expression of a gene involved in neurodevelopment and psychiatric risk (*BDNF*) have been linked with childhood trauma. Given the intergenerational patterns of health and behavior, and previous links between childhood trauma and *BDNF* methylation and expression, this study investigated the potential for maternal history of traumatic experiences to influence development in her newborn, via changes in her newborn’s *BDNF* methylation and expression. We found that mothers’ trauma history was associated with epigenetic regulation of *BDNF* in their newborns. Moreover, the association between maternal trauma and *BDNF* methylation and expression patterns were moderated by newborn sex. Male newborns showed increased *BDNF* expression with maternal exposure to child abuse (*p* = .001), and increased *BDNF* methylation with greater maternal fear (*p* = .001). Female newborns showed reduced *BDNF* expression with greater maternal fear (*p* = .004). Practitioners strive to identify prevention and intervention avenues that will reduce the harmful effects of trauma. Future research should consider the potential for maternal historical trauma experiences to influence offspring DNA methylation and gene expression in a manner that could alter development and inform novel prevention strategies.

## Introduction

Trauma, an experience that elicits fear, exerts significant influence on mental and physical health throughout the lifespan and is associated with intergenerational patterns of development and health (Doucet & Rovers, 2010; Fargas-Malet & Dillenburger, 2016; Schwerdtfeger & Goff, 2007; Yehuda et al., 2000). As childhood is a period with more plasticity in development and growth, individual adaptations to trauma in childhood can become more easily embedded in the biological systems still under development (Van der Kolk, 2003).

### Biological embedding of social stress

The associations between childhood trauma, fear, and physical and mental health can best be explained as the process that allows for our adaptations to the environment to be reflected in our cells during early development (Perry, 2009). These biological adaptations can result from a range of environments such as nurturing or threatening and have been found to manifest intergenerationally (Perry, 2009; Brand et al., 2010). One example of a biological adaptation associated with childhood trauma and fear is the sensitization of the hypothalamus-pituitary-adrenal (HPA) axis (Perry, 2009; Van der Kolk, 2003). A child born with a sensitized HPA axis may be more vulnerable to the deleterious effects of maltreatment and other adversities due to the influence of persistent stress activation during times of rapid neurodevelopment (Perry, 2009). Moreover, a sensitized HPA axis has been linked to psychopathology, including major depression (Heim et al., 2008). Consideration of the biological embedding of social stress, such as childhood trauma, helps us understand more about the potential for a childhood trauma to exert influence on human development from mother to child.

Investigations considering intergenerational associations often are rooted in the hypothesis of fetal programming. Fetal programming is a process of biological embedding during prenatal development that can generate significant alterations in behavior, physical, and mental health (Kapoor et al., 2006). Maternal physical and mental health during pregnancy drives fetal programming, generating a combination of risk and resilience factors within the newborn (Conradt et al., 2015; Conradt et al., 2013; Lillycrop & Burdge, 2015). The interaction between the biological embedding of trauma and fetal programming provide a framework, generally, of how intergenerational transmission of trauma can occur.

### DNA methylation and gene expression

Epigenetic mechanisms, like DNA methylation, are involved in our developmental adaptations to our experiences, such as trauma. DNA methylation is a structural modification to DNA that can regulate expression of a gene or change the magnitude of its activity without changing the DNA sequence. This process plays an integral role in development, health, and behavior by enabling tissue and context specific expression from a single genetic code (Bagot et al., 2014). Childhood trauma has been linked with patterns of DNA methylation and expression of a gene involved in processes of brain development, *BDNF* (Brain Derived Neurotrophic Factor), previously associated with a variety of adult psychopathology (D’Addario et al., 2012; Fuchikami et al., 2011; Thaler et al., 2014). Although DNA methylation can alter gene expression, methylation does not always correlate with changes in expression (Jones, 2012). Therefore, expression data was included in this study to develop a more comprehensive picture of gene regulation patterns.

### Gaps in the knowledge: human development and the central nervous system

Childhood trauma has been associated with patterns of DNA methylation and expression of genes involved in the central nervous system development such as glucocorticoid receptor and brain development genes (Blaze, Asok & Roth, 2015; Oberlander et al., 2008; Weder et al., 2014). *BDNF* is one such gene known for its role in neurogenesis and synaptogenesis and is a good candidate to investigate for intergenerational transmission of trauma (Perroud et al., 2013). Animal models have shown *BDNF* gene regulation patterns associated with fear memory consolidation, and fear is an integral part of the fight or flight response to traumatic events (Peters et al., 2010; Lubin, Roth & Sweatt, 2008; Perry, 2006). Rodent research has found an intergenerational link between childhood trauma and DNA methylation of *BDNF* and *GR* genes (Oberlander et al., 2008; Roth et al., 2009). Oberlander et al. (2008) identified differential GR methylation in newborn cord blood according to maternal depressed mood during pregnancy. However, human models have not explored the potential for childhood trauma and fear in the mother to transfer influence on gene regulation of *BDNF* in newborns (Blaze & Roth, 2015). DNA methylation patterns on *BDNF* have been linked to multiple types of psychopathology such as bipolar disorder and major depression representing potential mental and physical health risk for newborns if *BDNF* gene regulation is influenced by maternal trauma (D’Addario et al., 2012; Fuchikami et al., 2011; Thaler et al., 2014). However, gene regulation pathways through which trauma exerts intergenerational influences are just being discovered (Yehuda et al., 2015). DNA methylation, and gene expression, can change in response to our experiences, especially during early development (Bagot & Meaney, 2010). Therefore, current interventions could exert effects on a molecular level with the potential to influence developmental trajectories and outcomes. The more we understand about the effects of maternal trauma experience on infant gene regulation, the more opportunities we can identify to intervene for improved quality of life for mothers and their children. Thus, this study investigated the influence of maternal trauma history and associated fear on newborn *BDNF* methylation and expression in umbilical cord blood.

## Methods

### Sample and procedures

This study utilizes data from a larger longitudinal investigation of the Conditions Affecting Neurocognitive Development and Learning in Early Childhood (CANDLE) study, housed at the University of Tennessee Health Sciences Center. The authors assert that all procedures contributing to this work comply with the ethical standards of the relevant national guidelines on human experimentation outlined in the Belmont Report, and with the Helsinki Declaration of 1975, as revised in 2008, and was approved by the UTK Institutional Review Board (UTK IRB-15-02534-XM). Low medical risk maternal participants in the CANDLE study were recruited from local prenatal clinics in the Memphis metropolitan area and enrolled during pregnancy. All adult participants signed informed consent documents and participants under eighteen years of age provided a parent signed informed consent document. Participants were followed through pregnancy and delivery, and the mother-child pairs are currently being followed until middle childhood. Selection criteria included maternal age between 16 and 40 years, singleton and low-risk pregnancy. A broad range of trauma experiences and other psychosocial symptoms were assessed.

The current study uses a group of volunteers from the CANDLE cohort who agreed to cord blood collection after birth to allow for biological investigations of the effects of the perinatal environment on child development. This subsample has been used in previous studies to investigate DNA methylation and gene expression (Adkins, Tylavsky & Krushkal, 2012). DNA and RNA were extracted from umbilical cord blood, and DNA methylation was measured by CANDLE researchers as previously described, and gene expression was measured by CANDLE researchers as previously described (Adkins et al., 2011a; Adkins et al., 2011b; Adkins, Tylavsky & Krushkal, 2012; Schroeder et al., 2011). We assessed the cord blood DNA methylation for contamination by maternal blood using the umbilical cord blood Y chromosomes as a predictor for newborn sex and all subjects were correctly matched. We selected *BDNF* for candidate gene analysis because of previous research that linked stress and fear with patterns of *BDNF* methylation and expression. Our analyses focused on DNA methylation of the two available positions in the *BDNF* gene (cg16257091 and cg27351358) measured with the HumanMethylation27 array (Illumina Inc.), as well as *BDNF* expression (ILMN_1809543) measured with the Human WG-6 expression array (Illumina Inc.). The BDNF CpG sites for DNA methylation are important because they are located in the CpG island (11:27699782-27701489) and have previously been shown with differential methylation in associations with stress or fear (Kertes et al., 2017; Roth et al., 2009).

### Measures

Traumatic experiences were assessed using responses from the Traumatic Life Events Questionnaire (TLEQ; Kubany et al., 2000). This 23-item self-report measure covers 20 types of traumatic events, including childhood abuse, warfare, domestic violence, and natural disasters. For each item respondents indicate if the event occurred (0 = no, 1 = yes) and if the event resulted in “intense fear, helplessness, or horror” in response (0 = no, 1 = yes). Two trauma exposure variables were computed. Respondents were prompted in item Q21 to identify what event was the most distressful of the queried 20 trauma experiences. The trauma events selected were dichotomized to represent “interpersonal”/“non-interpersonal” (IPT/NIPT). Childhood abuse (CAb) was summed from items identifying physical abuse before age 13 years (Q12) and identifying sexual abuse before age 13 years (Q15). The CAb score was then dichotomized to differentiate mothers who reported childhood physical or sexual abuse from mothers who did not (no/yes). The presence or absence of fear was retrospectively reported for each traumatic event (0 = no, 1 = yes), and a summary score was computed as a lifetime trauma fear score (LTF), with higher values indicating more fear in relation to the reported traumatic experience(s). The TLEQ fear summary score has been evaluated for psychometric properties and determined to be an adequate fear measure (Johnson et al., 2010; Kubany et al., 2000). Furthermore, self-report of fear has been shown to be as reliable and valid as objective situational measures (Lanyon & Manosevitz, 1966).

The TLEQ has been found to have overall reliability with multiple populations, as well as good content validity as assessed by an expert panel, and good discriminant validity (Kubany et al., 2000). Furthermore, the TLEQ is considered an accepted trauma measure, and it has been used for comparison to assess newly established brief trauma measures (Gray et al., 2004).

### DNA methylation

Umbilical cord blood was collected by hospital staff following standard procedures and the whole blood was used to extract and measure DNA methylation and gene expression. Umbilical cord blood is one tissue previously used in multiple studies investigating DNA methylation in offspring (Boeke et al., 2012; Finer et al., 2015; Liu et al., 2014; Morales et al., 2014). The DNA extraction was completed by CANDLE researchers at the Department of Health and Science in the University of Tennessee at Memphis using the Wizard Genomic DNA purification kit (Promega Corp.). The EZ-96 DNA Methylation kit was used to bisulphite-convert the DNA (Zymo Research). Candidate gene methylation status was measured using the Infinium HumanMethylation27 Bead chip (Illumina Inc.) according to manufacturer’s protocol. Two *BDNF* CpG sites were identified for DNA methylation analyses. ComBat was used to control for variation among the chips that could confound results (Johnson, Li & Rabinovic, 2007). The new adjusted data set was used for all methylation analyses.

### Gene expression

We utilized the Human WG-6 expression array (Illumina Inc.) to measure gene expression, and one *BDNF* probe was selected for this study. The gene expression data underwent standard quality control (QC) procedures to identify low quality data for removal from analyses. First, data were screened to determine what genes were expressed. Detected *p*-values greater than .01 were set to missing values as standard in expression data and considered not detected. Samples with less than 10% of the gene probes detected were eliminated. Probes with less than 10% of the samples detected were eliminated. Second, we normalized the data for analysis by completing quantile normalization, scaled the data, and completed log2 transformation.

### Statistical analyses

This investigation employed linear regression to examine main effects of maternal report of IPT, CAb, and LTF on DNA methylation and gene expression of *BDNF* in the newborn. *BDNF * methylation is assessed as the proportion of methylated to unmethylated DNA strands at each measured CpG site (cg16257091, cg27351358) resulting in a value ranging 0-1. *BDNF* expression is a continuous value reflecting the level of RNA transcripts specific to *BDNF* (ILMN_1809543).

Mediation analyses were conducted in R version 1.1.383 using the Psych package to assess indirect effects of maternal trauma. Methylation and expression values were analyzed in the R package MethLAB, controlling for the following covariates: newborn sex, newborn race, and cell composition (Kilaru et al., 2012; Revelle, 2017; R Core Team, 2013). Finally, as multiple studies have shown that childhood adversity can generate different epigenetic patterns based on sex, after examining the combined newborn cohort, we stratified the sample by newborn sex to determine if and how relationships might vary between males and females (Doherty, Forster & Roth, 2016; Roth et al., 2014).

## Results

There were slightly more African American (55%) compared to White (45%) participants, and African American mothers were younger (25.0  ± 5.2 years) than Caucasian mothers (28.6  ± 4.6 years; *p* < .0001). However, there was no difference according to race in the trauma predictors IPT/NIPT, or CAb. Furthermore, no multicollinearity was observed between IPT/NIPT, and CAb (Tolerance = .85, .99), and correlations were small (*r* =  − .08, .17).

### Combined-sex analyses

The two maternal trauma variables (IPT/NIPT, CAb) and maternal fear (LTF) were assessed for degree of relatedness. IPT/NIPT significantly predicted LTF (*p* = .047), though it only accounted for 1.5% of the variation. CAb accounted for the largest proportion of variance (17%) in LTF (*p* < .001, *B* = 2.36).

There was no association between IPT/NIPT and newborn *BDNF* methylation or expression (*p* > .05). CAb did not associate with *BDNF* methylation or expression in the full cohort (*p* > .05). LTF did not reach statistical significance as a mediator of IPT/NIPT or CAb with *BDNF* methylation and expression in the full cohort.

### Sex-stratified analyses

**Figure 1:**
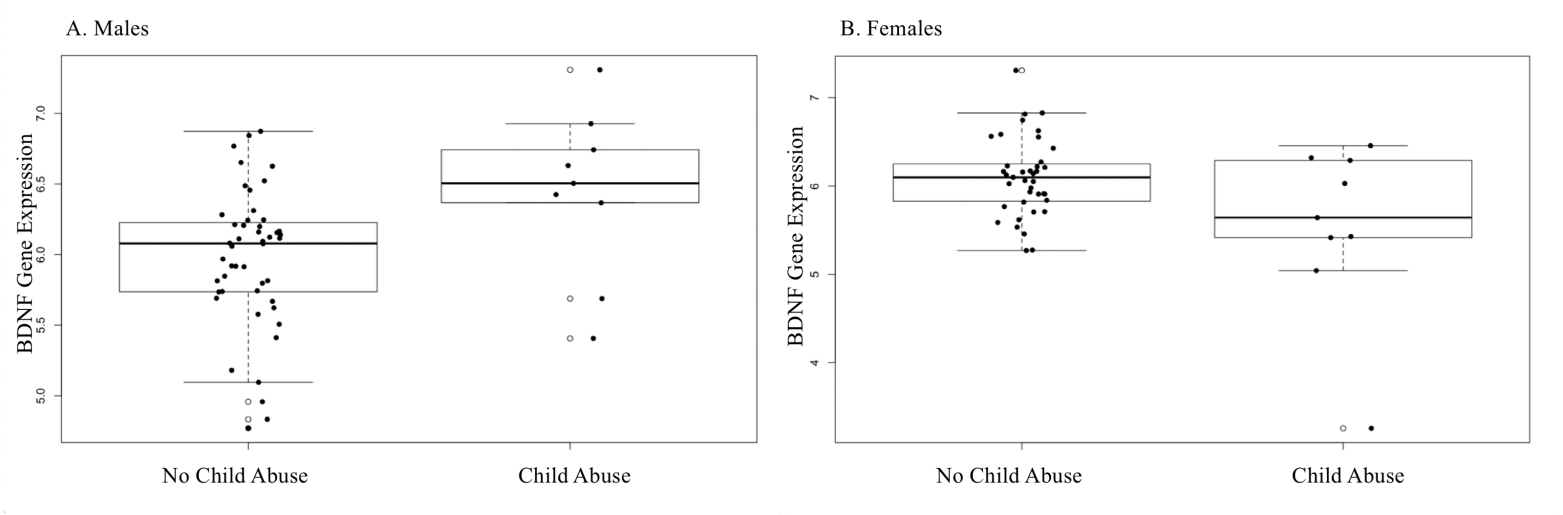
CAb and BDNF gene expression sex-stratified. (A) CAb (No Child abuse, Child abuse) and BDNF gene expression in male newborns. Males born to mothers who experienced childhood abuse showed increased expression of BDNF (B = .004, *p* = .001). *The bottom error bar for child abuse in the males’ plot is at the same value as the bottom of the boxplot line. (B) CAb showed reduced BDNF expression in female newborns, although the association did not reach statistical significance (B = −.123, *p* = .05).

IPT/NIPT did not vary significantly between newborn sexes (*p* > .05). However, mothers of female newborns were more likely to report a history of child abuse (*t* = 2.12, *p* = .04) *B* = .116, bootstrap CI [.003–.228]. There were no significant differences in *BDNF* methylation based on CAb in either sex (cg16257091- .2% ± 1.6% for males and .1% ± 1.7% for females) (cg27351358- .4% ± 1.9% for males and .4% ± 1.6% for females). CAb associated with increased *BDNF* expression in male newborns as seen in Fig. 1 (group *M* = 6.44, *SD* = .59, *p* = .001) *B* = .471, bootstrap CI [.056–.865]. Among females, the association with *BDNF* expression was much more attenuated, although it approached significance (group *M* = 5.54, *SD* = .98, *p* = .050). The absence of a significant association in females, combined with a small effect size for males likely contributed to the lack of a significant association in the combined cohort for CAb.

**Figure 2:**
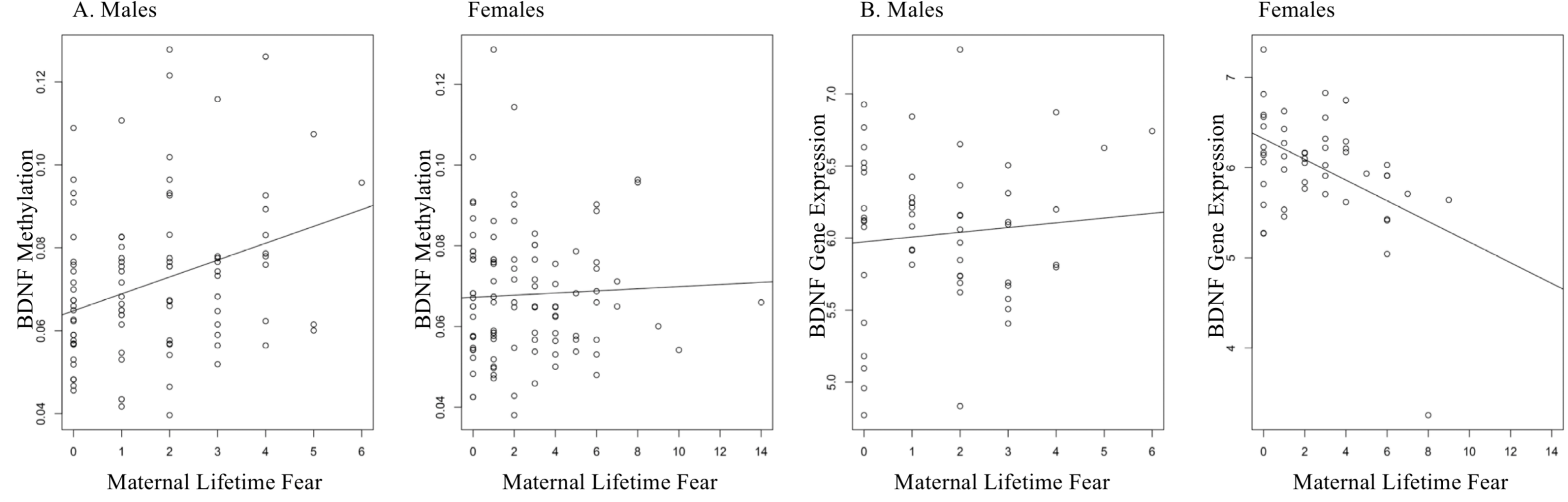
LTF and BDNF DNAm and gene expression. (A) A positive association between LTF and BDNF DNA methylation in males in the promotor region of the gene (B = .004, M = 1.62, *p* = .001). (B) LTF and BDNF DNA methylation in females (*p* > .05). (C) LTF and BDNF gene expression in males (*p* > .05). (D) A negative association emerged for LTF and BDNF expression in female newborns (B = −.123, M = 2.72, *p* = .004).

LTF also differed between the newborn sexes (*t* = 3.24, *p* = .006) *B* = .995, bootstrap CI [.41–1.58], with mothers of females showing more variation in fear. LTF associated with higher *BDNF* methylation of the promotor region in male newborns (*B* = .004, *M* = 1.62, *SD* = 1.52, *p* = .001, bootstrap CI [.001–.006]), but it did not correlate with *BDNF* expression. LTF did not associate with *BDNF* expression in males (*p* = .50). Female newborns showed a negative association between LTF and *BDNF* expression (*B* = −.123, *M* = 2.72, *SD* = 2.47, *p* = .004, bootstrap CI [−.222–−.038]), yet it did not correlate with *BDNF* methylation. LTF and *BDNF* methylation did not associate in females (*p* = .38). It is not uncommon to find an absence of correlation between DNA methylation and gene expression because there are additional mechanisms that can influence gene expression (Phillips, 2008). Figure 2 displays *BDNF* methylation and gene expression for males and females.

**Figure 3:**
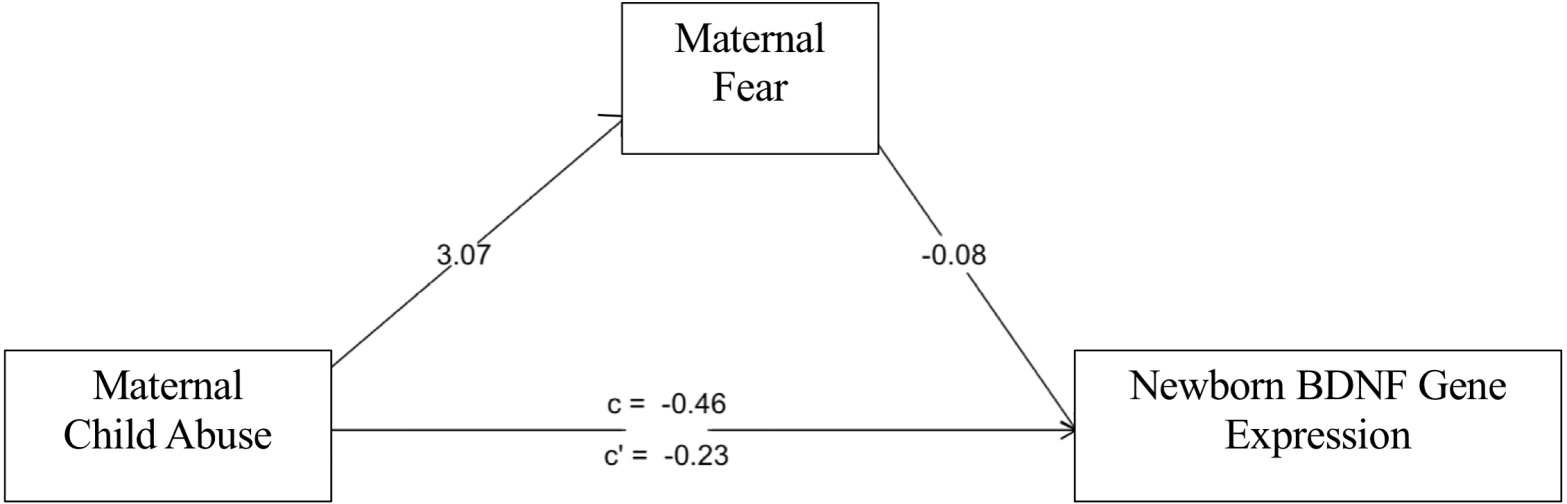
Fear mediation model. LFT mediates effects of CAb on *BDNF* expression in female newborns.

The sex-stratified mediation analyses revealed a sex-specific nature of the path of influence as modeled in Fig. 3. IPT/NIPT did not reach significance in the mediation model for male or female *BDNF* methylation or expression. However, in female newborns, CAb associated with LTF (*B* = 3.07, *p* = 2 × 10^−8^) which associated with *BDNF* expression (B = −.08, *p* = .001) showing joint significance (Kenny & Judd, 2014). Bootstrap confidence intervals were calculated as a second test of significance for the mediated effect of CAb on *BDNF* expression (B = −.22, bootstrap CI [−.52–−.01], R2 = .19). CAb did not show a mediated effect in male newborns for *BDNF* expression.

## Conclusion

This study investigated trauma in a multidimensional approach and identified associations according to trauma types. This is important given the rise in research focusing on adverse childhood experiences (ACEs) that utilizes a count measure for exposure to specific events considered stressful and/or traumatic (Felitti et al., 1998). Our findings showed mother’s exposure to childhood abuse associated with newborn gene expression (albeit in a sex-specific manner) whereas whether the trauma was of an interpersonal nature did not. Moreover, fear proved to be an important mechanism for child abuse exposure to exert influence from mother to child. The absence of significant associations with many of the trauma analyses could be a reflection of the complex nature of trauma and the difficulty in measuring the abstract concept. Future research would benefit from including a fear measure in trauma investigations to help reduce potential type II error.

Our results suggest child abuse exposure and lifetime trauma-related fear in one generation can affect gene regulation in offspring that could influence early development. Furthermore, this study identified direct and indirect pathways for child abuse to exert influence from mother to child. Including fear in the mediation model allowed a glimpse at how certain experiences are more likely to be associated with higher fear responses across the lifetime, such as childhood physical or sexual abuse, that maternal fear mediates some of the effects of maternal trauma for female newborns, and that maternal fear exerts direct influence of its own on newborn *BDNF* gene regulation in a sex-specific manner.

The sex-specific nature of the relationships identified in this study is consistent with previous research showing that childhood trauma generates different epigenetic patterns between the sexes (Doherty, Forster & Roth, 2016; Roth et al., 2014). This could be due to females’ developing under different neuroendocrine conditions in utero because males undergo a masculinization of the brain that is triggered by DNA methylation (Nugent & McCarthy, 2011).

The associations with regulation of the *BDNF* gene is of interest because of the known involvement in human development, most notably neurogenesis and synaptogenesis, and links to psychopathology. For example, the *BDNF* epigenetic profile of prenatally stressed mice has shown similar epigenetic signatures as the post-mortem brain tissues of human patients with schizophrenia (Dong et al., 2015). However, analyzing *BDNF* methylation and expression in umbilical cord blood does not permit the knowledge of what is manifested in brain tissue, but it does allow identification of associations with predictors and outcomes. For example, prior research using human blood showed increased *BDNF* methylation associated with increased suicidal thoughts and behavior, and a literature review considering research conducted with brain and blood tissue identified abnormal *BDNF* signaling as a biomarker for increased suicide risk (Kang et al., 2013; Dwivedi, 2010). In our study, we found maternal fear positively associated with *BDNF* methylation in male newborns. This is not to suggest that these male newborns will develop suicidal thoughts and behavior, though they may be at increased risk. In a recent rodent model, rodents exposed to early life stress showed increased BDNF methylation in both sexes, and decreased BDNF expression in females (Coley et al., 2019). Of interest is the offspring of those early life stress rodents also showed increased BDNF methylation similar to the intergenerational findings of this study (Coley et al., 2019). These findings can add to the growing knowledge of intergenerational effects of early life stress and fear. Although many of the studies are animal models, some research has shown human mothers during pregnancy who reported early life stress have increased brain derived neurotrophic factor in their amniotic fluid which could be affecting fetus development and gene regulation (Deuschle et al., 2018).

There are some limitations to this study. This investigation was conducted on one small subset of a population sample in an urban community of west Tennessee, which may not be generalizable to other regions. Furthermore, trauma exposure and fear experiences were based on self-report, and emotional stimulation is often triggered with remembering traumatic events which can influence reporting (Perrott et al., 1998; Summit, 1983). It is also possible that pregnant women with a history of childhood abuse could be prone to experience greater pregnancy stress and we did not have data to assess this within our cohort. Although it is still uncertain how these gene regulation patterns are specifically reflected in the brain, proxy tissue is still the least invasive and most ethical approach to investigations with living humans. Though we could have employed a genome-wide approach, we chose a hypothesis-driven evaluation of BDNF because we were underpowered to detect associations after accounting for multiple comparisons. Finally, though BDNF has many CpG sites whose methylation could be tested for association, we focused on the two located on the CpG island for which there was available data measured by the HumanMethylation27K array.

It is also important to consider the benefits of investigating DNA methylation and gene expression in social science research. Epigenetic mechanisms may be able to provide insight into trauma-related symptoms resistant to treatment. Social scientists are making strong arguments for a need to acknowledge, educate about, and participate in inquiries when possible to examine epigenetic mechanisms, given the potential influence on human function and development (Combs-Orme, 2013). Moreover, generational patterns of distressing trauma-related behavior and psychological health symptoms have long been examined in interest of identifying new prevention opportunities. The influence of fear-evoking experiences on development, behavior, and subsequent generations has been an area of intrigue, offering insight into potential pathways for generational psychological health symptoms such as anxiety and depression. DNA methylation and gene expression may help us identify poor outcome pathways for individuals who experience adversities such as childhood trauma. This is important for practitioners who are, by trade, engaged in helping populations suffering chronic discrimination, oppression, poverty, and violence.

The potential for adverse experience to influence subsequent generations should be investigated further. Future research would benefit from inclusion of other prenatal variables that might be influenced by trauma exposure also would help refine the amount of variance accounted for by the prenatal environment. Some variables that can greatly influence the prenatal environment include the nutrient balance of the mother’s diet, household income, and social connectedness. These variables could be linked to trauma based upon previous research that found childhood abuse associations with poorer employment status and therefore lower income (Barrett, Kamiya & Sullivan, 2014). Ultimately, this pursuit may provide new avenues for prevention and interventions to improve quality of life for children, adults, and families overall.

##  Supplemental Information

10.7717/peerj.8858/supp-1 Supplemental Information 1 Code

10.7717/peerj.8858/supp-2 Supplemental Information 2 Secondary Data Retrieved from CANDLE for analyses

## Additional Information and Declarations

### Data Availability

The following information was supplied regarding data availability: The code file is available in the Supplemental Files and includes all of the analyses conducted in R for this article.

## References

[ref-1] Adkins RM, Krushkal J, Tylavsky FA, Thomas F (2011a). Racial differences in gene-specific DNA methylation levels are present at birth. Birth Defects Research Part A: Clinical and Molecular Teratology.

[ref-2] Adkins RM, Thomas F, Tylavsky FA, Krushkal J (2011b). Parental ages and levels of DNA methylation in the newborn are correlated. BMC Medical Genetics.

[ref-3] Adkins RM, Tylavsky FA, Krushkal J (2012). Newborn umbilical cord blood DNA methylation and gene expression levels exhibit limited association with birth weight. Chemistry & Biodiversity.

[ref-4] Bagot RC, Labonte B, Pena CJ, Nestler EJ (2014). Epigenetic signaling in psychiatric disorders: stress and depression. Dialogues in Clinical Neuroscience.

[ref-5] Bagot RC, Meaney MJ (2010). Epigenetics and the biological basis of gene × environment interactions. Journal of the American Academy of Child & Adolescent Psychiatry.

[ref-6] Barrett A, Kamiya Y, Sullivan VO (2014). Childhood sexual abuse and later-life economic consequences. Journal of Behavioral and Experimental Economics.

[ref-7] Blaze J, Asok A, Roth TL (2015). The long-term impact of adverse caregiving environments on epigenetic modifications and telomeres. Frontiers in Behavioral Neuroscience.

[ref-8] Blaze J, Roth TL (2015). Evidence from clinical and animal model studies of the long-term and transgenerational impact of stress on DNA methylation. Seminars in Cell & Developmental Biology.

[ref-9] Boeke CE, Baccarelli A, Kleinman KP, Burris HH, Litonjua AA, Rifas-Shiman SL, Tarantini L, Gillman M (2012). Gestational intake of methyl donors and global LINE-1 DNA methylation in maternal and cord blood: prospective results from a folate-replete population. Epigenetics.

[ref-10] Brand SR, Brennan PA, Newport DJ, Smith AK, Weiss T, Stowe ZN (2010). The impact of maternal childhood abuse on maternal and infant HPA axis function in the postpartum period. Psychoneuroendocrinology.

[ref-11] Coley EJL, Demaestri C, Ganguly P, Honeycutt JA, Peterzell S, Rose N, Ahmed N, Holschbach M, Trivedi M, Brenhouse HC (2019). Cross-generational transmission of early life stress effects on HPA regulators and Bdnf are mediated by sex, lineage, and upbringing. Frontiers in Behavioral Neuroscience.

[ref-12] Combs-Orme T (2013). Epigenetics and the social work imperative. Social Work.

[ref-13] Conradt E, Fei M, LaGasse L, Tronick E, Guerin D, Gorman D, Marsit CJ, Lester BM (2015). Prenatal predictors of infant self-regulation: the contributions of placental DNA methylation of NR3C1 and neuroendocrine activity. Frontiers in Behavioral Neuroscience.

[ref-14] Conradt E, Lester BM, Appleton AA, Armstrong DA, Marsit CJ (2013). The roles of DNA methylation of NR3C1 and 11 *β*-HSD2 and exposure to maternal mood disorder in utero on newborn neurobehavior. Epigenetics.

[ref-15] D’Addario C, Dell’Osso B, Palazzo MC, Benatti B, Lietti L, Cattaneo E, Galimberti D, Fenoglio C, Cortini F, Scarpini E, Arosio B, Di Francesco A, Di Benedetto M, Romualdi P, Candeletti S, Mari D, Bergamaschini L, Bresolin N, Maccarrone M, Altamura AC (2012). Selective DNA methylation of BDNF promoter in bipolar disorder: differences among patients with BDI and BDII. Neuropsychopharmacology.

[ref-16] Deuschle M, Hendlmeier F, Witt S, Rietschel M, Gilles M, Sánchez-Guijo A, Fañanas L, Hentze S, Wudy SA, Hellweg R (2018). Cortisol, cortisone, and BDNF in amniotic fluid in the second trimester of pregnancy: effect of early life and current maternal stress and socioeconomic status. Development and Psychopathology.

[ref-17] Doherty TS, Forster A, Roth TL (2016). Global and gene-specific DNA methylation alterations in the adolescent amygdala and hippocampus in an animal model of caregiver maltreatment. Behavioural Brain Research.

[ref-18] Dong E, Dzitoyeva SG, Matrisciano F, Tueting P, Grayson DR, Guidotti A (2015). Brain-derived neurotrophic factor epigenetic modifications associated with schizophrenia-like phenotype induced by prenatal stress in mice. Biological Psychiatry.

[ref-19] Doucet M, Rovers M (2010). Generational trauma, attachment, and spiritual/religious interventions. Journal of Loss and Trauma.

[ref-20] Dwivedi Y (2010). Brain-derived neurotrophic factor and suicide pathogenesis. Annals of Medicine.

[ref-21] Fargas-Malet M, Dillenburger K (2016). Intergenerational transmission of conflict-related Trauma in Northern Ireland: a behavior analytic approach. Journal of Aggression Maltreatment & Trauma.

[ref-22] Felitti V, Anda R, Nordenberg D, Williamson D, Spitz A, Edwards V, Koss M, Marks J (1998). Relationship of childhood abuse and household dysfunction to many of the leading causes of death in adults: the adverse childhood experiences (ACE) study. American Journal of Preventive Medicine.

[ref-23] Finer S, Mathews C, Lowe R, Smart M, Hillman S, Foo L, Sinha A, Williams D, Rakyan VK, Hitman GA (2015). Maternal gestational diabetes is associated with genome-wide DNA methylation variation in placenta and cord blood of exposed offspring. Human Molecular Genetics.

[ref-24] Fuchikami M, Morinobu S, Segawa M, Okamoto Y, Yamawaki S, Ozaki N, Inoue T, Kusumi I, Koyama T, Tsuchiyama K, Terao T (2011). DNA methylation profiles of the Brain-Derived Neurotrophic Factor (BDNF) gene as a potent diagnostic biomarker in major depression. PLOS ONE.

[ref-25] Gray MJ, Litz BT, Hsu JL, Lombardo TW (2004). Psychometric properties of the life events checklist. Assessment.

[ref-26] Heim C, Newport DJ, Mletzko T, Miller AH, Nemeroff CB (2008). The link between childhood trauma and depression: insights from HPA axis studies in humans. Psychoneuroendocrinology.

[ref-27] Johnson CS, Heffner JL, Blom TJ, Anthenelli RM (2010). Exposure to traumatic events among treatment-seeking, alcohol dependent women and men without PTSD. Journal of Traumatic Stress.

[ref-28] Johnson WE, Li C, Rabinovic A (2007). Adjusting batch effects in microarray expression data using empirical Bayes methods. Biostatistics.

[ref-29] Jones PA (2012). Functions of DNA methylation: islands, start sites, gene bodies and beyond. Nature Reviews Genetics.

[ref-30] Kang HJ, Kim JM, Lee JY, Kim SY, Bae KY, Kim SW, Shin IS, Kim HR, Shin MG, Yoon JS (2013). BDNF promoter methylation and suicidal behavior in depressive patients. Journal of Affective Disorders.

[ref-31] Kapoor A, Dunn E, Kostaki A, Andrews MH, Matthews SG (2006). Fetal programming of hypothalamo-pituitary-adrenal function: prenatal stress and glucocorticoids. The Journal of Physiology.

[ref-32] Kenny DA, Judd CM (2014). Power anomalies in testing mediation. Psychological Science.

[ref-33] Kertes DA, Bhatt SS, Kamin HS, Hughes DA, Rodney NC, Mulligan CJ (2017). BNDF methylation in mothers and newborns is associated with maternal exposure to war trauma. Clinical Epigenetics.

[ref-34] Kilaru V, Barfield R, Schroeder J, Smith A, Conneely K (2012). MethLAB: a GUI package for the analysis of array-based DNA methylation data. Epigenetics.

[ref-35] Kubany ES, Leisen MB, Kaplan AS, Watson SB, Haynes SN, Owens JA, Burns K (2000). Development and preliminary validation of a brief broad-spectrum measure of trauma exposure: the Traumatic Life Events Questionnaire. Psychological Assessment.

[ref-36] Lanyon RI, Manosevitz M (1966). Validity of self-reported fear. Behaviour Research and Therapy.

[ref-37] Lillycrop KA, Burdge GC (2015). Maternal diet as a modifier of offspring epigenetics. Journal of Developmental Origins of Health and Disease.

[ref-38] Liu X, Chen Q, Tsai HJ, Wang G, Hong X, Zhou Y, Zhang C, Liu C, Liu R, Wang H (2014). Maternal preconception body mass index and offspring cord blood DNA methylation: exploration of early life origins of disease. Environmental and Molecular Mutagenesis.

[ref-39] Lubin FD, Roth TL, Sweatt JD (2008). Epigenetic regulation of BDNF gene transcription in the consolidation of fear memory. Journal of Neuroscience.

[ref-40] Morales E, Groom A, Lawlor DA, Relton CL (2014). DNA methylation signatures in cord blood associated with maternal gestational weight gain: results from the ALSPAC cohort. BMC Research Notes.

[ref-41] Nugent BM, McCarthy MM (2011). Epigenetic underpinnings of developmental sex differences in the brain. Neuroendocrinology.

[ref-42] Oberlander TF, Weinberg J, Papsdorf M, Grunau R, Misri S, Devlin AM (2008). Prenatal exposure to maternal depression, neonatal methylation of human glucocorticoid receptor gene (NR3C1) and infant cortisol stress responses. Epigenetics.

[ref-43] Perrott K, Morris E, Martin J, Romans S (1998). Cognitive coping styles of women sexually abused in childhood: a qualitative study. Child Abuse and Neglect.

[ref-44] Perroud N, Salzmann A, Prada P, Nicastro R, Hoeppli ME, Furrer S, Ardu S, Krejci I, Karege F, Malafosse A (2013). Response to psychotherapy in borderline personality disorder and methylation status of the BDNF gene. Translational Psychiatry.

[ref-45] Perry BD (2006). Fear and learning: trauma-related factors in the adult education process. New Directions for Adult and Continuing Education.

[ref-46] Perry BD (2009). Examining child maltreatment through a neurodevelopmental lens: clinical applications of the neurosequential model of therapeutics. International Perspectives on Stress & Coping.

[ref-47] Peters J, Dieppa-Perea LM, Melendez LM, Quirk GJ (2010). Induction of fear extinction with hippocampal-infralimbic BDNF. Science.

[ref-48] Phillips T (2008). The role of methylation in gene expression. Nature Education.

[ref-49] R Core Team (2013). https://www.R-project.org/.

[ref-50] Revelle WR (2017). https://CRAN.R-project.org/package=psych.

[ref-51] Roth TL, Lubin FD, Funk AJ, Sweatt JD (2009). Lasting epigenetic influence of early-life adversity on the BDNF gene. Biological Psychiatry.

[ref-52] Roth TL, Matt S, Chen K, Blaze J (2014). Bdnf DNA methylation modifications in the hippocampus and amygdala of male and female rats exposed to different caregiving environments outside the homecage. Developmental Psychobiology.

[ref-53] Schroeder JW, Conneely KN, Cubells JF, Kilaru V, Newport DJ, Knight BT, Stowe ZN, Brennan PA, Krushkal J, Tylavsky FA (2011). Neonatal DNA methylation patterns associate with gestational age. Epigenetics.

[ref-54] Schwerdtfeger KL, Goff BSN (2007). Intergenerational transmission of trauma: exploring mother–infant prenatal attachment. Journal of Traumatic Stress.

[ref-55] Summit RC (1983). The child sexual abuse accomodation syndrome. Child Abuse & Neglect.

[ref-56] Thaler L, Gauvin L, Joober R, Groleau P, De Guzman R, Ambalavanan A, Israel M, Wilson S, Steiger H (2014). Methylation of BDNF in women with bulimic eating syndromes: associations with childhood abuse and borderline personality disorder. Progress in Neuro-Psychopharmacology and Biological Psychiatry.

[ref-57] Van der Kolk BA (2003). The neurobiology of childhood trauma and abuse. Child and Adolescent Psychiatric Clinics of North America.

[ref-58] Weder N, Zhang H, Jensen K, Yang BZ, Simen A, Jackowski A, Lipschitz D, Douglas-Palumberi H, Ge M, Perepletchikova F, O’Loughlin K, Hudziak JJ, Gelernter J, Kaufman J (2014). Child abuse, depression, and methylation in genes involved with stress, neural plasticity, and brain circuitry. Journal of the American Academy of Child & Adolescent Psychiatry.

[ref-59] Yehuda R, Bierer LM, Schmeidler J, Aferiat DH, Breslau I, Dolan S (2000). Low cortisol and risk for PTSD in adult offspring of holocaust survivors. American Journal of Psychiatry.

[ref-60] Yehuda R, Daskalakis NP, Bierer LM, Bader HN, Klengel T, Holsboer F, Binder EB (2015). Holocaust exposure induced intergenerational effects on FKBP5 methylation. Biological Psychiatry.

